# Supplementary material for: Nutrient supply controls the linkage between species abundance and ecological interactions in marine bacterial communities
Source: Nat Commun. 2022 Jan 10;13:175. doi: 10.1038/s41467-021-27857-6 (PMC8748817; doi:10.1038/s41467-021-27857-6)
Supplement: Supplementary file 1 — Supplementary Information File [file 41467_2021_27857_MOESM1_ESM.pdf]

**Supplementary Information**

# Nutrient supply controls the linkage between species abundance and ecological interactions in marine bacterial communities

Tianjiao Dai[1,2], Donghui Wen[2*], Colin T. Bates[3], Linwei Wu[3], Xue Guo[1], Suo Liu[1], Yifan Su[1], Jiesi Lei[1], Jizhong Zhou[3,4,5], and Yunfeng Yang[1*]

[1]State Key Joint Laboratory of Environment Simulation and Pollution Control, School of Environment, Tsinghua University, Beijing, China

[2]College of Environmental Sciences and Engineering, Peking University, Beijing 100871, China

[3]Institute for Environmental Genomics and Department of Microbiology and Plant Biology, University of Oklahoma, Norman, Oklahoma

[4]School of Civil Engineering and Environmental Sciences, University of Oklahoma, Norman, OK, USA

[5]Earth and Environmental Sciences, Lawrence Berkeley National Laboratory, Berkeley, California

[*]To whom correspondence may be addressed. Email: dhwen@pku.edu.cn; Tel & Fax: +86-010-62751923; and yangyf@tsinghua.edu.cn; Tel: +86-010-62784692; Fax: +86-010-62794006;

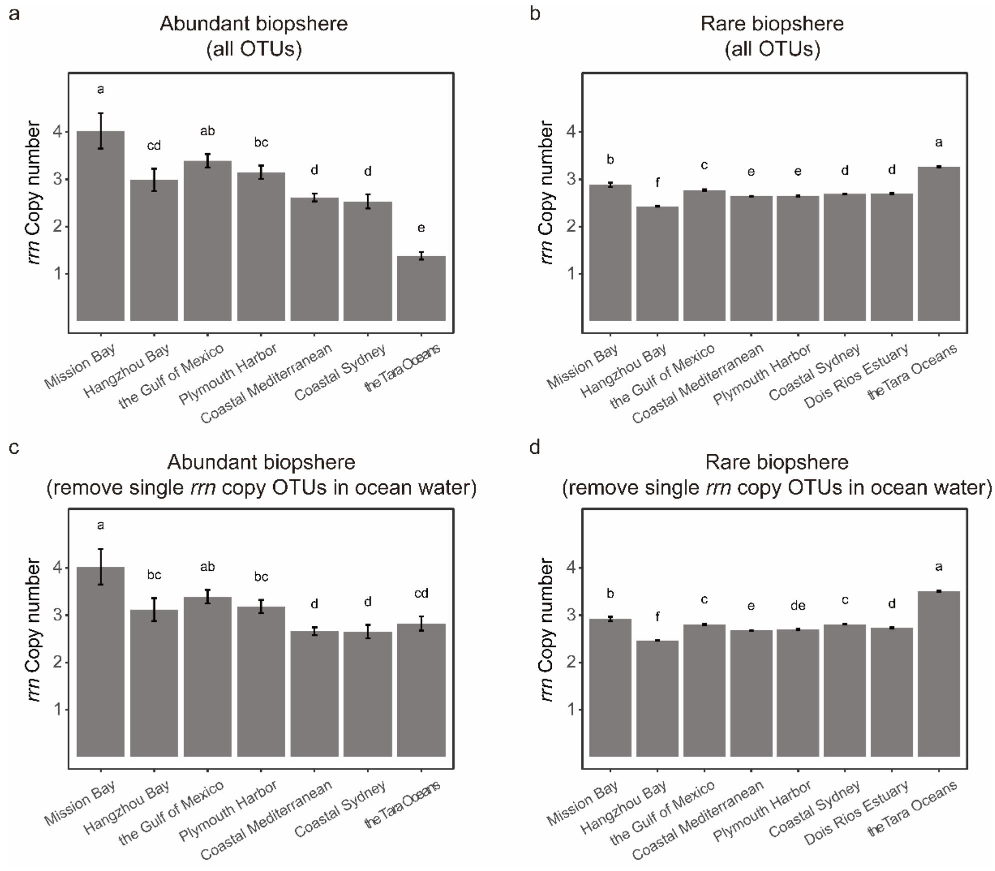


**Supplementary Figure 1 The *rrn* copy numbers of abundant and rare biosphere OTUs.** All OTUs are included in panels **a** and **b,** while OTUs with a single *rrn* copy in the ocean water dataset are removed in panels **c** and **d**. Bars represent mean values, error bars represent the standard errors, and lowercase letters above the bars indicate significant differences based on one-way ANOVA followed by LSD test (adjusted $P < 0.05$ by Bonferroni method).The sample sizes are listed in Supplementary Table 1. In panels **a** and **c**, the bar representing Dois Rios estuary is missed because no OTU in the dataset was classified as abundant biosphere based on the criteria we defined.

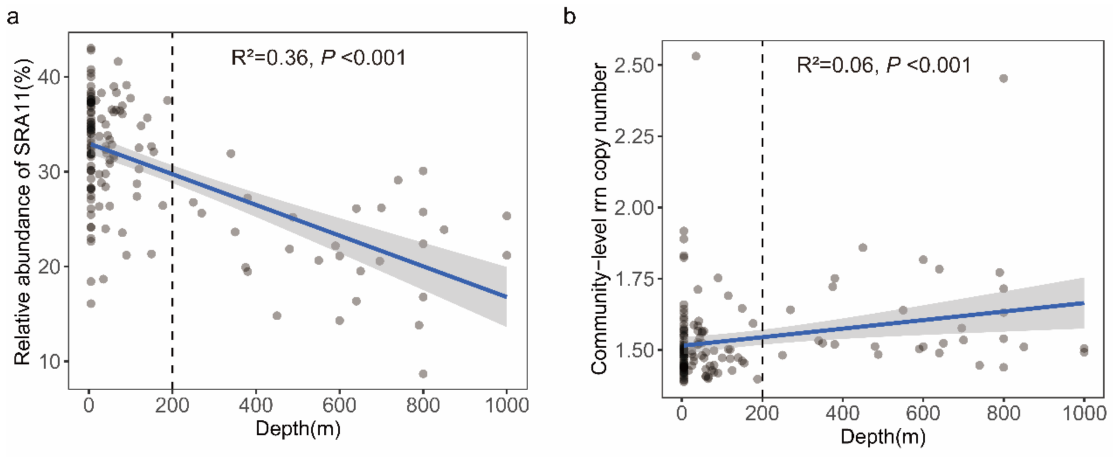


**Supplementary Figure 2 The relative abundance of SAR11 and the community community-level *rrn* copy number from the surface to the deep ocean.** The dashed lines in **a** and **b** divide the surface ocean (0 - 200 m) and the deep ocean (200 - 1000 m). Adjusted $R^2$ and $P$ values from linear regression (blue line) are shown together with 95% confidence intervals (gray region).

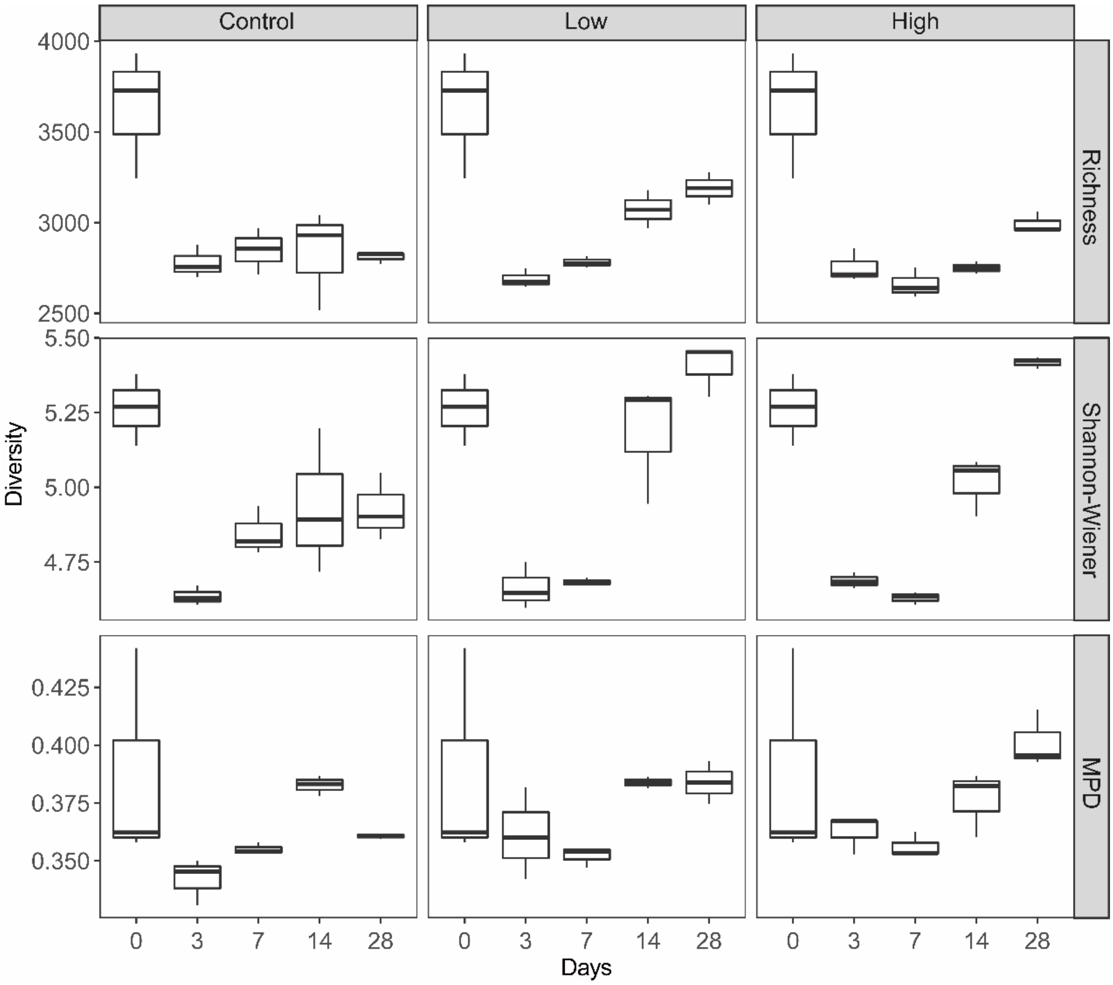


**Supplementary Figure 3 Dynamics of bacterial communities' taxonomic (measured by richness and Shannon-Weiner index) and phylogenetic (measured by mean pairwise distance, MPD) diversity in response to different nutrient supplies.** In the boxplots, hinges indicate the 25th, 50th, and 75th percentiles, whiskers indicate 1.5 × interquartile ranges (n = 3 biologically independent samples).

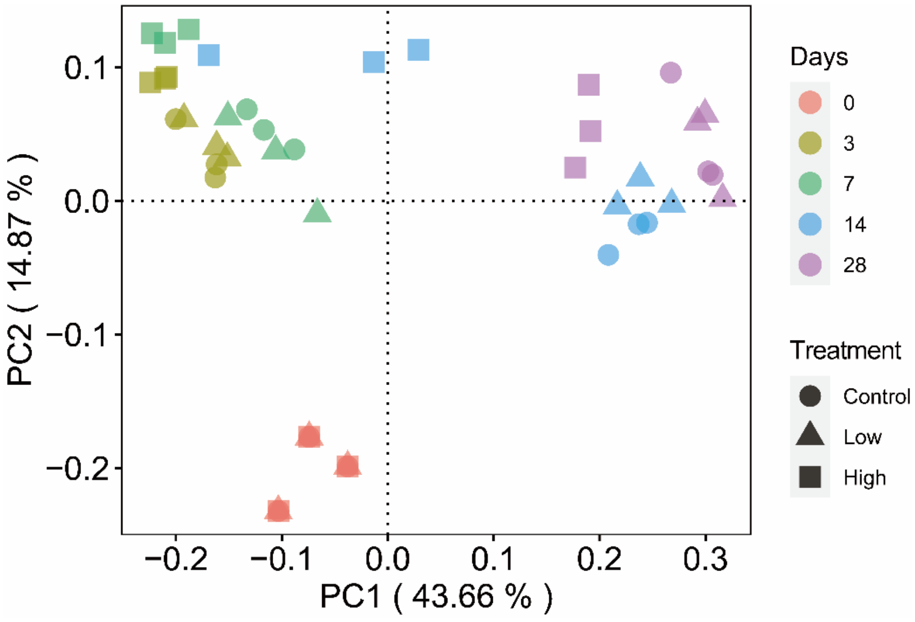


**Supplementary Figure 4 Principle coordinates ordination based on Bray-Curtis distances showing changes in bacterial community composition by different nutrient supplies.**

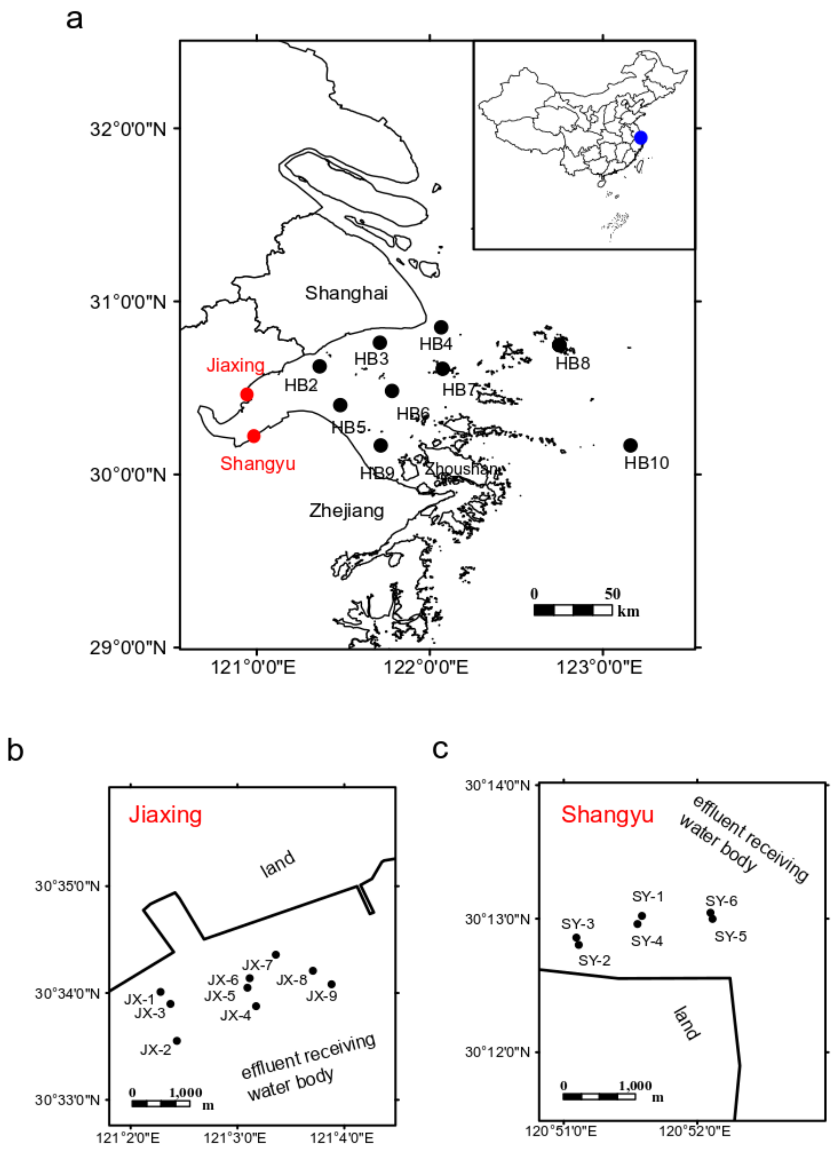


**Supplementary Figure 5 Sampling sites in coastal Hangzhou Bay, China. a**, sampling sites inner Hangzhou Bay (HB). **b** and **c**, sampling sites in the coastal wastewater receiving area near Jiaxing City (JX) and Shangyu City (SY).

**Supplementary Table 1 The *rrn* copy numbers of the OTUs belonging to abundant, intermediate, and rare biospheres.**

| | Datasets | No. of Samples | No. of OTUs | [a]*rrn* copy number (OTUs %) | | | |
|---|---|---|---|---|---|---|---|
| | | | | Abundant | Intermediate | Rare | [b]Overall |
| ***Coastal sediment*** | Mission Bay | 20 | 1759 | 4.02±0.37**a** (n = 11) | 3.01±0.06**b** (n = 668) | 2.88±0.04**b** (n = 1080) | 2.89±0.09**a** |
| | Hangzhou Bay | 72 | 14424 | 2.99±0.24**a** (n = 48) | 2.61±0.04**b** (n = 1344) | 2.43±0.01**c** (n =13032) | 2.18±0.01**d** |
| | the Gulf of Mexico | 6 | 5839 | 3.39±0.14**a** (n = 86) | 3.07±0.05**b** (n = 654) | 2.77±0.02**c** (n = 5153) | 2.51±0.01**b** |
| | Plymouth Harbor | 65 | 21225 | 3.15±0.14**a** (n = 62) | 2.97±0.04**a** (n = 1289) | 2.64±0.01**b** (n = 19874) | 2.45±0.02**b** |
| | Coastal Mediterranean | 11 | 7343 | 2.62±0.08**b** (n = 123) | 2.80±0.06**a** (n = 527) | 2.65±0.01**b** (n = 6693) | 2.34±0.01**c** |
| | Coastal Sydney | 60 | 89355 | 2.53±0.15**a** (n = 42) | 2.72±0.03**a** (n = 1291) | 2.69±0.01**a** (n = 88022) | 2.24±0.01**d** |

| | | | | | | | |
|---|---|---|---|---|---|---|---|
| ***Ocean water*** | Dois Rios Estuary | 9 | 6500 | NA (n = 0) | 2.82±0.04**a** (n = 987) | 2.70±0.02**b** (n = 5513) | 2.26±0.07**cd** |
| | the *Tara* Oceans | 139 | 26961 | 1.38±0.07**c** (n = 105) | 2.93±0.04**b** (n = 2445) | 3.26±0.01**a** (n = 24411) | 2.18±0.01**d** |

[a]the *rrn* copy numbers of OTUs are presented as mean ± s.e., where lowercase letters in bold indicate significant difference based on one-way ANOVA followed by LSD test, and data in the brackets indicate the proportion of OTUs in the dataset.

[b]the average *rrn* copy number of all OTUs in a community, i.e., the abundance unweighted community-level average *rrn* copy number.

**Supplementary Table 2 Pearson's correlation (two-sided) between environmental nutrients and the community-level *rrn* copy number.**

| Nutrients | Overall | | Abundant | | Intermediate | | Rare | |
|---|---|---|---|---|---|---|---|---|
| | r | *P* | r | *P* | r | *P* | r | *P* |
| **Hangzhou Bay, China** | | | | | | | | |
| [a]Ammonia (mg/L) | 0.508 | <0.001 | 0.523 | <0.001 | 0.404 | 0.001 | 0.300 | 0.018 |
| [a]Phosphate (mg/L) | 0.410 | 0.001 | 0.432 | 0.001 | 0.251 | 0.052 | 0.128 | 0.356 |
| [b]Total phosphorus (mg/kg) | 0.491 | <0.001 | 0.513 | <0.001 | 0.357 | 0.004 | 0.198 | 0.143 |
| **Coastal Sydney** | | | | | | | | |
| [b]TOC (mg/g) | 0.765 | <0.001 | 0.532 | <0.001 | 0.737 | <0.001 | 0.707 | <0.001 |
| [b]TN (%) | 0.779 | <0.001 | 0.542 | <0.001 | 0.734 | <0.001 | 0.754 | <0.001 |
| **Coastal Mediterranean** | | | | | | | | |
| [b]Silt and clay (%) | 0.782 | 0.013 | 0.803 | 0.013 | 0.704 | 0.023 | -0.732 | 0.021 |
| **the Tara Oceans** | | | | | | | | |
| [a]Phosphate (μmol/L) | 0.635 | <0.001 | 0.317 | <0.001 | 0.488 | <0.001 | 0.518 | <0.001 |
| [a]Nitrate and Nitrite (μmol/L) | 0.643 | <0.001 | 0.272 | 0.002 | 0.489 | <0.001 | 0.499 | <0.001 |

[a]measured for water;

[b]measured for sediment.

**Supplementary Table 3 Effect of nutrient availability and phylogenetic structure on community-level *rrn* copy number by partial Mantel test[a].**

| | Datasets | Nutrients | | Phylogenetic structure | |
|---|---|---|---|---|---|
| | | r | *P* | r | *P* |
| *Coastal sediment* | Hangzhou Bay, China | 0.220 | 0.026 | 0.751 | 0.001 |
| | Coastal Sydney | 0.406 | 0.001 | 0.266 | 0.001 |
| | Coastal Mediterranean | 0.602 | 0.001 | -0.229 | 0.947 |
| *Ocean water* | the Tara Oceans | 0.130 | 0.029 | 0.483 | 0.001 |

[a]Changes in the phylogenetic structure are measured by weighted Unifrac distance, and changes in nutrients availability are measured by Euclidean distance. The statistics were derived from partial Mantel test based on two-sided Pearson's correlation.

**Supplementary Table 4 Topological features of bacterial communities' association networks in the coastal sediments and global ocean water.**

| Topological properties | Hangzhou Bay | Plymouth | Coastal Sydney | the Tara Oceans |
|---|---|---|---|---|
| Correlation coefficient cutoff | 0.87 | 0.89 | 0.89 | 0.88 |
| No. of nodes | 368 | 262 | 485 | 1158 |
| No. of links | 530 | 514 | 869 | 7217 |
| Negative links (%) | 31.13 | 80.35 | 14.38 | 0.04 |
| $R^2$ of power-law | 0.959 | 0.828 | 0.945 | 0.829 |
| Average degree | 2.88 | 3.924 | 3.583 | 12.465 |
| Average clustering coefficient | 0.371 (0.147±0.019)[a] | 0.114 (0.111±0.020) | 0.223 (0.139±0.016) | 0.657 (0.589±0.006) |
| Average path length (avgGD) | 8.08 (4.894±0.149) | 3.566 (3.596±0.064) | 6.104 (4.656±0.192) | 4.052 (4.485±0.097) |
| Transitivity | 0.337 (0.137±0.015) | 0.064 (0.054±0.007) | 0.229 (0.167±0.010) | 0.554 (0.526±0.003) |

| | | | | |
|---|---|---|---|---|
| Modularity | 0.841<br>(0.703±0.015) | 0.587<br>(0.524±0.010) | 0.68<br>(0.578±0.015) | 0.755<br>(0.707±0.004) |

[a]Data in the brackets represent the topological properties of 100 random networks (mean ± sd), which were generated by rewiring all the links with the identical numbers of nodes and links to the corresponding empirical network.

**Supplementary Table 5 Quantitative effects of nutrients supply, succession time, and their interactions on the microcosm bacterial community variations by permutational multivariate analysis of variance (adonis)[a].**

| Source of Variation | Levels | F.Model | $R^2$ | *P* |
|---|---|---|---|---|
| Time (Days) | 0, 3, 7, 14, 28 | 14.827 | 0.153 | 0.001 |
| Nutrients | Control<br>Low<br>High | 4.598 | 0.095 | 0.001 |
| Time*Nutrients | | 1.801 | 0.037 | 0.046 |

[a]Permutational multivariate analysis of variance (adonis) was based on Bray-Curtis dissimilarity distance. The two-way ANOVA model was set as dissimilarity ~ nutrients × time using function *adonis* in the R package "*vegan*".

**Supplementary Table 6 Classifications of bacterial OTUs in the microcosm experiments.**

| Treatment | Total OTUs | Abundant | Intermediate | Rare |
|---|---|---|---|---|
| Control | 10334 | 75 | 156 | 10103 |
| Low | 10719 | 74 | 163 | 10482 |
| High | 10418 | 88 | 148 | 10182 |

**Supplementary Table 7 Pearson's correlation (two-sided) between nutrient concentrations and community-level *rrn* copy number in the microcosm experiment.**

| Treatments | Ammonia | | Phosphate | |
|---|---|---|---|---|
| | r | *P* | r | *P* |
| Control | 0.616 | 0.033 | 0.397 | 0.201 |
| Low | 0.718 | 0.009 | 0.614 | 0.034 |
| High | 0.865 | < 0.001 | 0.878 | <0.001 |

**Supplementary Table 8 Topological features of microcosm bacterial association networks.**

| Topological properties | Control | Low | High |
|---|---|---|---|
| Correlation coefficient cutoff | 0.913 | 0.910 | 0.898 |
| No. of nodes | 1036 | 1301 | 1328 |
| No. of links | 1392 | 3797 | 2188 |
| $R^2$ of power-law | 0.961 | 0.903 | 0.935 |
| Average degree | 2.687 | 5.837 | 3.295 |
| Average clustering coefficient | 0.156 (0.105±0.0)[a] | 0.194 (0.173±0.004) | 0.130 (0.102±0.00) |
| Average path length | 7.132 (6.502±0.1) | 5.834 (5.381±0.134) | 7.798 (6.778±0.12) |
| Transitivity | 0.237 (0.165±0.0) | 0.338 (0.307±0.003) | 0.280 (0.225±0.00) |
| Modularity | 0.784 (0.746±0.0) | 0.551 (0.496±0.010) | 0.737 (0.701±0.00) |

[a]Data in the brackets represent the topological properties of 100 random networks (mean ± sd), which were generated by rewiring all the links with the identical numbers of nodes and links to the corresponding empirical network.

**Supplementary Table 9 Proportion of negative associations in networks for microcosm bacterial communities[a].**

| Association pairs | Control | Low | High |
|---|---|---|---|
| Abundant - Abundant | 33.3% (60) | 45.7% (129) | 51.5% (101) |
| Intermediate - Intermediate | 46.4% (28) | 37.9% (140) | 49.1% (57) |
| Rare - Rare | 71.4% (840) | 60.7% (1790) | 73.6% (1306) |
| Abundant - Intermediate | 42.3% (71) | 43.3% (298) | 46.2% (119) |
| Abundant - Rare | 44.3% (192) | 46.6% (672) | 49.0% (304) |
| Intermediate - Rare | 39.3% (201) | 43.5% (768) | 46.2% (301) |

[a]The proportion of negative associations was calculated as negative links/total links that identified for each association pairs in the network, and the data in bracket indicates the absolute number of total links.
