# Supplementary material for: Nutrient supply controls the linkage between species abundance and ecological interactions in marine bacterial communities
Source: Nat Commun. 2022 Jan 10;13:175. doi: 10.1038/s41467-021-27857-6 (PMC8748817; doi:10.1038/s41467-021-27857-6)
Supplement: Supplementary file 3 — Description of Additional Supplementary Files [file 41467_2021_27857_MOESM3_ESM.docx]

**Description of Additional Supplementary Files**

File Name: Supplementary Data 1

Description: List of the bacterial 16S rRNA gene sequence data accession number and associated metadata collected from other studies except for the *Tara* Oceans data which is available from the companion website (see “Data Availability”).
